# Initial experience in staging primary oesophageal/gastro-oesophageal cancer with 18F-FDG PET/MRI

**DOI:** 10.1186/s41824-021-00117-y

**Published:** 2021-12-13

**Authors:** Amy R. Sharkey, Bert-Ram Sah, Samuel J. Withey, Shaheel Bhuva, Radhouene Neji, Sami Jeljeli, Adrian Green, Gary J. R. Cook, Vicky Goh, On behalf of the Guy’s & St Thomas’ Oesophago-Gastric Research Group, C. R. Baker, F. Chang, S. Chicklore, M. Cominos, A. Coombes, A. R. Davies, S. George, B. Gill-Barman, J. N. Dunn, J. A. Gossage, N. Griffin, M. Hill, O. Hynes, C. Iezzi, A. Jacques, M. Kelly, U. Mahadeva, N. Maisey, R. McEwan, J. Meenan, S. Ngan, K. Owczarczyk, A. Qureshi, A. Reyhani, M. Subesinghe, G. Tham, J. Waters, S. S. Zeki

**Affiliations:** 1 Department of Cancer Imaging, School of Biomedical Engineering and Imaging Sciences, King’s College London, London, UK; 2 Department of Radiology, Guy’s and St Thomas’ NHS Foundation Trust, London, UK; 3 Department of Diagnostic, Interventional, and Pediatric Radiology, Inselspital, University of Bern, Bern, Switzerland; 4 King’s College London and Guy’s and St Thomas’ PET Centre, St Thomas’ Hospital, London, UK; 5 MR Research Collaborations, Siemens Healthcare, Frimley, UK

**Keywords:** PET/CT, PET/MR, Oesophageal cancer, Gastro-oesophageal cancer

## Abstract

**Background:**

^18^F-fluorodeoxyglucose positron emission tomography/magnetic resonance imaging (18F-FDG PET/MRI) may improve cancer staging by combining sensitive cancer detection with high-contrast resolution and detail. We compared the diagnostic performance of 18F-FDG PET/MRI to ^18^F-fluorodeoxyglucose positron emission tomography/computed tomography (18F-FDG PET/CT) for staging oesophageal/gastro-oesophageal cancer. Following ethical approval and informed consent, participants with newly diagnosed primary oesophageal/gastro-oesophageal cancer were enrolled. Exclusions included prior/concurrent malignancy. Following 324 ± 28 MBq 18F-FDG administration and 60-min uptake, PET/CT was performed, immediately followed by integrated PET/MRI from skull base to mid-thigh. PET/CT was interpreted by two dual-accredited nuclear medicine physicians and PET/MRI by a dual-accredited nuclear medicine physician/radiologist and cancer radiologist in consensus. Per-participant staging was compared with the tumour board consensus staging using the McNemar test, with statistical significance at 5%.

**Results:**

Out of 26 participants, 22 (20 males; mean ± SD age 68.8 ± 8.7 years) completed 18F-FDG PET/CT and PET/MRI. Compared to the tumour board, the primary tumour was staged concordantly in 55% (12/22) with PET/MRI and 36% (8/22) with PET/CT; the nodal stage was concordant in 45% (10/22) with PET/MRI and 50% (11/22) with PET/CT. There was no statistical difference in PET/CT and PET/MRI staging performance (*p* > 0.05, for T and N staging). The staging of distant metastases was concordant with the tumour board in 95% (21/22) with both PET/MRI and PET/CT. Of participants with distant metastatic disease, PET/MRI detected additional metastases in 30% (3/10).

**Conclusion:**

In this preliminary study, compared to 18F-FDG PET/CT, 18F-FDG PET/MRI showed non-significant higher concordance with T-staging, but no difference with N or M-staging. Additional metastases detected by 18F-FDG PET/MRI may be of additive clinical value.

**Supplementary Information:**

The online version contains supplementary material available at 10.1186/s41824-021-00117-y.

## Background

Oesophageal cancer, including cancer extending to the gastro-oesophageal junction, remains a leading cause of cancer death worldwide (Enzinger and Mayer 2003). Late presentation results in poor overall survival, with 5-year survival rates ranging from 15 to 25% (Pennathur et al. 2013). Curative treatment combines neoadjuvant chemoradiotherapy with surgery, while palliative treatment options include chemotherapy and local management, such as radiotherapy and endoscopic stenting (Pennathur et al. 2013; Hagen et al. 2012; Sjoquist et al. 2011). Optimal use of staging techniques is essential to predict prognosis and to tailor treatment to achieve the best possible outcomes.

Current clinical pathways incorporate contrast-enhanced computed tomography (CT) and 18F-fluorodeoxyglucose positron emission tomography/computed tomography (18F-FDG PET/CT) for staging patients planned for curative surgery (Lordick et al. 2016). Endoscopic ultrasound (EUS) may also be performed for very localised tumours, and some gastro-oesophageal cancers may undergo a staging laparoscopy. (In our centre, staging laparoscopy is performed in patients with evidence of subdiaphragmatic disease who is eligible for curative surgical resection following fitness assessment and initial staging investigations.) There are limitations to this staging approach; 18F-FDG PET/CT has a high sensitivity for metastatic disease; however, diagnostic accuracy remains suboptimal for locoregional staging (Vliet et al. 2008). Conversely, EUS has a high performance for local staging, but may not be successful in up to 45% of patients (Kelly et al. 2001).

MRI offers superior soft tissue contrast and high anatomical resolution, an advantage in locoregional and distant staging (Riddell et al. 2007a; Riddell et al. 2006; Riddell et al. 2007b). To date, exploration of the hybrid diagnostic utility of 18F-FDG PET/MRI in oesophageal cancer is limited. Lee et al. (2014) published a retrospective study from a sequential PET/MRI system, finding 18F-FDG PET/MRI demonstrated acceptable accuracy for T staging compared with EUS, and higher accuracy than EUS and 18F-FDG PET/CT for prediction of nodal staging, albeit at a statistically insignificant level. Linder et al. (2019) have more recently published a series of 16 patients undergoing 18F-FDG-PET/MRI for preoperative staging of oesophageal or gastro-oesophageal cancer, finding 18F-FDG-PET/MRI and 18F-FDG-PET/CT correlated well for most measured values with discrepancies mainly in the assessment of the T-stage.

We hypothesised that integrated 18F-FDG PET/MRI may streamline and improve staging compared to our current clinical practice by combining sensitive molecular imaging with high-contrast anatomical imaging. Therefore, the primary aim of our prospective study was to compare the diagnostic performance of integrated 18F-FDG PET/MRI for staging primary oesophageal/gastro-oesophageal cancers compared to our current clinical practice (contrast-enhanced CT and 18F-FDG PET/CT), with secondary aims of comparing the SUVmax in 18F-FDG PET/CT vs. 18F-FDG PET/MRI, and assessing correlations between ADCmean and corresponding SUVmax values.

## Materials and methods

### Participants

Research ethics committee approval and informed consent were obtained for this prospective single-centre feasibility study (Research Ethics Committee Number: 14/LO/0220). Twenty-six participants with newly diagnosed, histologically proven oesophageal/gastro-oesophageal cancer were eligible and enrolled from 2016 to 2018. Participants were excluded if there was a prior or concurrent malignancy and if they did not complete the 18F-FDG PET/MRI scan.

### Imaging

#### 18F-FDG PET/CT

Participants were injected with 324 ± 28 MBq 18F-FDG following a 4–6-h fast and after blood glucose levels were verified as ≤ 10.0 mmol/L. Imaging was performed on two identical scanners (Discovery 710, GE Healthcare, Chicago, IL, USA). Image acquisition was performed 60 min post-tracer injection from skull base to mid-thigh (5–8 bed positions; 3 min per bed position). PET images were reconstructed with a time-of-flight ordered subset expectation maximisation algorithm (2 iterations, 24 subsets) with a reconstructed slice thickness of 3.27 mm and pixel size 4.7 mm. The non-contrast CT component was acquired at 140 kVp with Smart mA (15–100 mA).

#### 18F-FDG PET/MRI

The average time period from the start of the 18F-FDG PET/CT to the start of the 18F-FDG PET/MRI was 52.5 ± 18.1 min. 18F-FDG PET/MRI was performed on an integrated system (Siemens Biograph mMR, Erlangen, Germany) straight after completion of the PET/CT acquisition. This was acquired from the skull base to mid-thigh (4–5 bed positions, 4 min per bed position). For each bed position, a two-point Dixon volume-interpolated breath-hold examination (VIBE) sequence was applied to derive an attenuation map (*u*-map) based on four tissue types: air, lung, soft tissue and fat. Other sequences per bed position included: T1-weighted Dixon VIBE, T2-weighted half-Fourier-acquired single-shot turbo spin-echo (HASTE) and free breathing diffusion-weighted sequences (DWI, *b* values of 50 and 900 s/mm^2^). Additional T1-weighted post-gadolinium contrast agent axial sequences of the liver and coronal sequence of the thorax were performed (Acquisition parameters: Additional file 1: Table S1). The PET images were reconstructed with a 3D iterative method (3 iterations, 21 subsets), with a voxel size of 2.3 × 2.3 × 5 mm, full width at half maximum of 4 mm and absolute scatter correction.

### Image interpretation

#### 18F-FDG PET/CT

18F-FDG PET/CT was interpreted by two dual-accredited nuclear medicine physicians in consensus. The location of the primary tumour and location and number of involved nodes (defined by FDG uptake or size, as per clinical practice) and distant metastases were recorded. A TNM stage was assigned for each participant according to the eighth edition of the American Joint Committee on Cancer TNM (tumour–node–metastasis) staging system (Rice et al. 2017). Primary tumour SUVmax was also measured.

#### 18F-FDG PET/MRI

18F-FDG PET/MRI image interpretation was performed by a dual-accredited nuclear medicine physician/radiologist and cancer radiologist in consensus, blinded to other imaging and outcome. Again, location of the primary tumour and location and number of involved nodes and distant metastases were recorded. The TNM stage was assigned for each participant. Primary tumour SUVmax and ADCmean value were also measured.

### Tumour board consensus staging

The final TNM stage, as documented by the tumour board consensus staging prior to treatment, was defined by all standard investigations, including imaging with contrast-enhanced CT, 18F-FDG PET/CT, EUS (where applicable) and staging laparoscopy (where applicable), but excluding 18F-FDG PET/MRI. Of note, EUS was not a suitable investigation for many of our patients and was only performed on 4/22 participants in this cohort, as many were ineligible due having an obstructing tumour which EUS scope could not traverse. The tumour board decision involved the consensus of cancer radiologists, nuclear medicine physicians, clinical and medical oncologists, upper gastrointestinal surgeons and other members of the multi-disciplinary team.

### Follow-up

Participants were followed up as per normal clinical practice, with a 3-year follow-up period detailed in this study.

### Statistical analysis

Per-participant diagnostic performance of 18F-FDG PET/MRI and 18F-FDG PET/CT was compared with tumour board staging set as the reference standard, using the McNemar test, with statistical significance at 5%.

The SUVmax recorded on 18F-FDG PET/MRI and 18F-FDG PET/CT was compared using the Wilcoxon signed-rank test.

Correlation between the ADCmean and SUVmax values on 18F-FDG PET/MRI was compared using Spearman’s rank-order correlation.

Further descriptive analysis was carried out on a case-by-case basis to determine whether the 18F-FDG PET/MRI study provided any additive information to the 18F-FDG PET/CT, especially for nodal or distant metastases.

## Results

### Participants

**Fig. 1:**
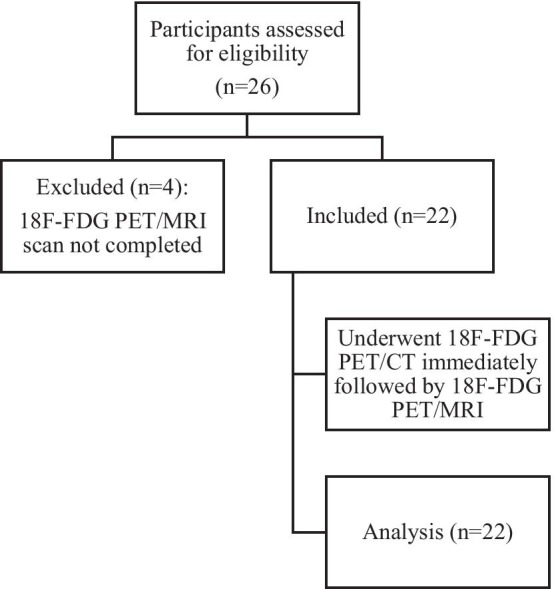
Participant flow diagram

Twenty-six participants with newly diagnosed oesophageal/gastro-oesophageal cancer were enrolled in this study. Out of 26 participants, 22 (20 males; 69 ± 9 years at time of 18F-FDG PET/CT imaging) underwent both standard investigations and 18F-FDG PET/MRI. Four out of 26 participants were excluded due to non-completion of 18F-FDG PET/MRI. The participant flow diagram is shown in Fig. 1.

Table 1 describes participant and tumour characteristics. Participants were followed up for 3 years, with 3/22 participants being lost to follow-up. Three out of 19 participants with follow-up remain alive and are disease-free, all of whom had a non-adenocarcinoma subtype (two squamous cell, one small cell), and thus predictably better outcomes.

**Table 1:** Participant and tumour characteristics, treatment and outcome

Participant characteristics	
Sex	20 Males (91%):2 females (9%)
Mean ± SD age (years)	68.8 ± 8.7
Tumour location	
Upper oesophagus	0
Mid-oesophagus	1
Distal oesophagus	8
gastro-oesophageal	13
Cancer type	
Adenocarcinoma	18
Squamous cell carcinoma	3
Small cell carcinoma	1
Treatment	
Neoadjuvant therapy + surgery	3
Definitive chemoradiotherapy	2
Surgery only	1
Palliative chemotherapy	7
Palliative radiotherapy ± chemotherapy	4
Declined treatment	1
Unknown	4
Outcome	
Alive	3
Deceased	16
Unknown	3

### Staging

18F-FDG PET/CT staging, 18F-FDG PET/MRI staging and tumour board consensus staging are presented in Table 2.

**Table 2:** Comparison of TNM staging for 18F-FDG PET/CT, 18F-FDG PET/MRI and tumour board consensus. Participant 13 had no increased tumour FDG uptake compared to background

Participant	18F-FDG PET/CT			18F-FDG PET/MRI			Tumour board consensus		
	T	N	M	T	N	M	T	N	M
1	3	3	1	3	3	1	3	2	1
2	3	1	1	4	1	1	3	1	0
3	2	2	1	2	1	1	3	2	1
4	2	2	0	3	2	0	3	2	0
5	2	0	0	3	0	0	3	0	0
6	2	2	0	4	1	0	3	1	0
7	2	1	0	3	1	0	4	1	0
8	3	1	0	3	1	0	3	1	0
9	2	0	0	2	0	0	3	0	0
10	3	2	0	3	2	0	3	1	0
11	2	3	1	3	3	1	3	2	1
12	3	1	0	3	1	0	3	2	0
13	3	1	0	3	1	0	3	1	0
14	2	1	0	4	2	0	3	1	0
15	3	3	0	3	3	0	4	2	0
16	3	2	1	3	3	1	3	1	1
17	2	3	1	2	3	1	3	3	1
18	2	2	1	3	1	1	3	3	1
19	2	1	1	2	1	1	3	2	1
20	2	0	0	2	0	0	3	1	0
21	3	2	1	4	2	1	4	3	1
22	4	2	1	4	2	1	4	2	1

#### T-staging

Using the tumour board consensus as a reference standard, there were T3 cancer in 18/22 (82%) participants and T4 cancer in 4/22 (18%) participants. The primary tumour stage was concordant with the tumour board consensus decision in 12/22 (54%) participants with 18F-FDG PET/MRI (over-staged 3, under-staged 7) and 8/22 (36%) participants with 18F-FDG PET/CT (over-staged 0, under-staged 14). There was no statistically significant difference between the modalities in terms of consensus with tumour board decision (*p* = 0.69).

#### N-staging

Using the tumour board consensus as a reference standard, 2/22 (9%) participants had a nodal stage of N0, 9/22 (41%) participants had N1, 8/22 (36%) participants had N2, and 3/22 (14%) participants had N3. The nodal stage was concordant with the tumour board consensus decision in 11/22 (50%) participants using 18F-FDG PET/CT (6 over-staged, 5 under-staged) and 10/22 (45%) participants using 18F-FDG PET/MRI (6 over-staged, 6 under-staged). Again, there was no statistically significant difference between the modalities in terms of consensus with the tumour board decision (*p* = 0.99).

#### M-staging

Using the tumour board consensus as a reference standard, 9/22 (41%) participants had distant metastases and 13/22 (59%) participants did not have distant metastases. The staging of metastatic spread was concordant with the tumour board consensus decision in 21/22 (95%) participants using 18F-FDG PET/CT (1 over-staged) and 21/22 (95%) participants using 18F-FDG PET/MRI (1 over-staged). Of note, the same participant was staged discordantly on both modalities, as both 18F-FDG PET/CT and 18F-FDG PET/MRI detected peritoneal disease which was not appreciated on the contrast-enhanced CT study.

### Comparison of SUVmax between 18F-FDG PET/CT and 18F-FDG PET/MRI

**Fig. 2:**
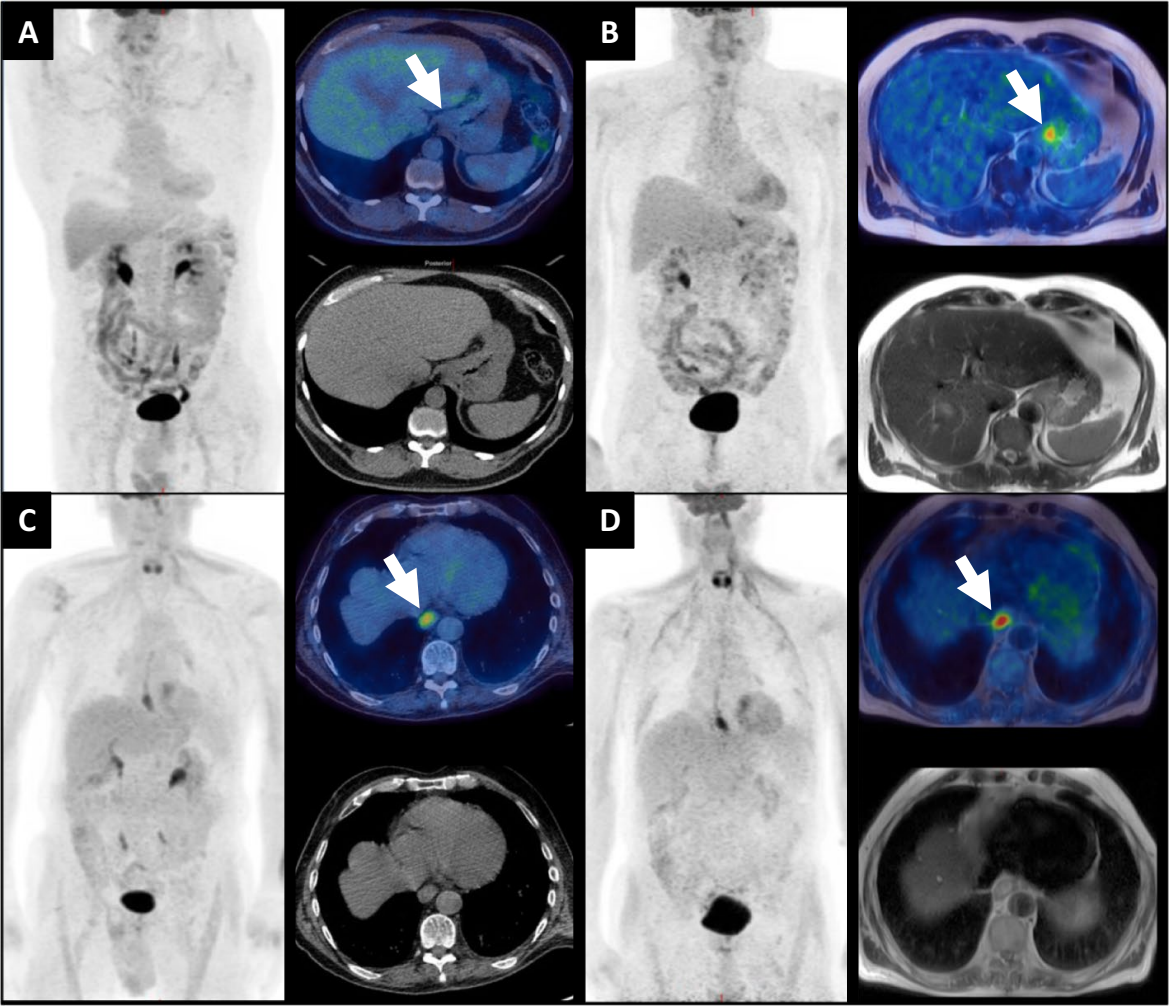
**a**, **b** A gastro-oesophageal adenocarcinoma, where uptake is noted on 18F-FDG PET/MRI (**b**) (highlighted by arrow), but lower corresponding uptake is noted on the 18F-FDG PET/CT (**a**). SUVmax was 9.4 on the 18F-FDG PET/MRI vs. 3.4 on the 18F-FDG PET/CT. **c** and **d** A lower oesophageal adenocarcinoma (highlighted by arrow), for which corresponding uptake is noted on both 18F-FDG PET/CT (**c**) and 18F-FDG PET/MRI (**d**). The SUVmax was 11.8 on the 18F-FDG PET/MRI vs. 7.2 on the 18F-FDG PET/CT

Table 3 summarises primary tumour SUVmax for 18F-FDG PET/CT and subsequent 18F-FDG PET/MRI for each participant. Comparative images are shown in Fig. 2. The average time from the start of the 18F-FDG PET/CT to the start of the 18F-FDG PET/MRI was 52.5 ± 18.1 min, noting that PET/CT acquisitions lasted 20 to 30 min depending on the number of bed positions and that participants subsequently emptied their bladders, transferred to the PET/MRI scanner in the same department and had coils fitted as part of the PET/MRI set-up. The reproducibility of SUVmax values between PET/CT and PET/MRI has been investigated by Ringheim et al. (2018). We feel the higher SUVmax values on 18F-FDG PET/MRI are likely primarily resulting from the increased time for uptake, although technical differences in the attenuation correction methods between PET/CT and PET/MRI could also have influenced differences. The average primary tumour SUVmax on PET/CT was 18.0 ± 12.4, versus an average SUVmax on PET/MRI of 22.5 ± 13.7 (Wilcoxon signed-rank test, *p* < 0.001).

**Table 3:** Primary tumour SUVmax for 18F-FDG PET/CT and PET/MRI and diffusion-weighted MRI mean apparent diffusion coefficient, ADCmean

Participant	PET/CT SUVmax	PET/MRI SUVmax	ΔSUVmax (%)	ADCmean (× 10^−3^mm^2^/s)
1	14.6	17.1	17	1.68
2	51.9	51.9	0	1.29
3	11.3	17.3	53	1.89
4	7.9	11.7	48	0.79
5	11.5	16.8	46	1.47
6	11.5	17.1	49	1.26
7	4	5.3	33	1.81
8	20.7	22.8	10	0.93
9	7.2	11.8	64	1.95
10	15.1	17	13	1.43
11	8.8	12.4	41	1.22
12	16.8	18.6	11	1.99
13	N/A	N/A	N/A	0.59
14	23.2	39.4	70	1.23
15	41.4	45.5	10	1.19
16	20.6	21.6	5	1.16
17	30	43.6	45	0.93
18	28.9	31.9	10	1.69
19	7.2	7.9	10	1.84
20	25.1	28.6	14	1.33
21	13.3	20.4	53	0.83
22	24.9	35.4	42	1.16

### Association between SUVmax and ADCmean with 18F-FDG PET/MRI

**Fig. 3:**
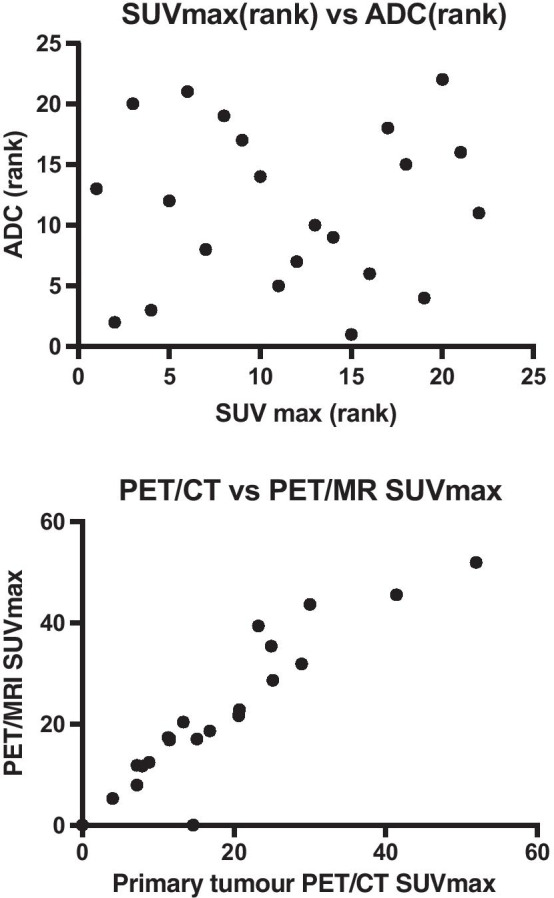
Graph showing the correlation between the 18F-FDG PET/MRI SUVmax and the diffusion-weighted apparent diffusion coefficient, ADC (top), with Bland–Altman plot of PET/CT SUVmax vs PET/MRI SUVmax (below)

Table 3 also summarises primary tumour mean apparent diffusion co-efficient (ADCmean) for each participant. There was no correlation between SUVmax and the corresponding ADCmean value, with a Spearman correlation coefficient of 0.05, *p* = 0.83, demonstrated in Fig. 3, which also demonstrates a Bland–Altman plot of the SUVmax values.

### Additional information with 18F-FDG PET/MRI

Differences in the imaging modalities are demonstrated in Fig. 3, showing differences in primary tumour uptake in a gastro-oesophageal adenocarcinoma on 18F-FDG PET/CT vs. 18F-FDG PET/MRI.

In terms of nodal evaluation, 18F-FDG PET/MRI did not improve nodal staging compared to 18F-FDG PET/CT. There was a difference between 18F-FDG PET/CT and 18F-FDG PET/MRI in 4/22 patients, with a lower number of nodes on 18F-FDG PET/MRI in 3/4 cases; in one case, this was due to bowel gas artefact obscuring the node.

**Fig. 4:**
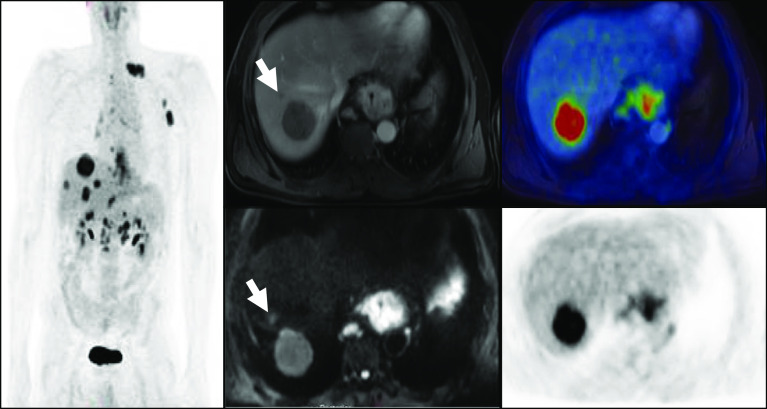
Oesophageal adenocarcinoma with additional liver metastases demonstrated by MRI. A non-FDG avid subcentimetre metastasis (highlighted by arrow) is demonstrated on diffusion-weighted and post-contrast-enhanced MRI, which was not detected by 18F-FDG PET/CT or contrast-enhanced CT

In 3/10 (30%) patients with distant metastases, more metastases were demonstrated on 18F-FDG PET/MRI compared to 18F-FDG PET/CT. In two cases, additional sites of peritoneal disease were demonstrated on 18F-FDG PET/MRI; in the third case, additional liver metastases were detected (Fig. 4). Although these findings are not captured in the comparative M staging (which offers only a binary 0 or 1), they could be important clinically and prognostically, suggesting that 18F-FDG PET/MRI may offer additional clinical benefit in some patients.

## Discussion

The primary tumour was concordantly staged with the tumour board consensus staging in 54% (12/22) of participants with 18F-FDG PET/MRI and 36% (8/22) with 18F-FDG PET/CT. Nodal staging was concordant with the tumour board consensus decision in 45% (10/22) with 18F-FDG PET/MRI and 50% (11/22) with 18F-FDG PET/CT. Our results show that within this small cohort, there is no statistically significant difference in T and N staging on 18F-FDG PET/CT and 18F-FDG PET/MRI, although 18F-FDG PET/MRI provides a non-significant higher concordance with the tumour board decision in terms of T staging, i.e. 54% (12/22) vs. 36% (8/22).

In terms of M staging, 18F-FDG PET/CT and 18F-FDG PET/MRI both exhibit 95% (21/22) concordance with tumour board decision, although, of note, 18F-FDG PET/MRI detected additional metastases in 3/10 participants with M1 disease. Comparing the staging to the tumour board consensus, the participants with discordant staging on 18F-FDG PET/CT tended to be the same participants with discordant staging on 18F-FDG PET/MRI. This is in keeping with other studies, also finding that 18F-FDG PET/MRI offers similar levels of diagnostic accuracy as 18F-FDG PET/CT; Linder et al. (2019) found that comparison of oesophageal tumour SUV measurements revealed strong correlations, without significant differences between 18F-FDG-PET/MRI or 18F-FDG-PET/CT.

Early studies showed disappointing results for the role of conventional MR imaging compared with CT in oesophageal cancer staging (Quint et al. 1985; Takashima et al. 1991), describing low accuracy in staging the tumours, mainly because of difficulty in detecting tumour invasion through the muscle layer into peri-oesophageal fat (Quint et al. 1985). However, MRI technology has advanced substantially since these initial studies. Furthermore, 18F-FDG PET/CT has emerged as a useful adjunct to conventional staging methods in oesophageal cancer and is of particular importance for the detection of unexpected distant metastases and recurrent disease (Rossum et al. 2015). Given the higher costs and lower availability of PET/MRI scanners, it is uncertain whether this technique could be performed routinely. However, as a combined modality, 18F-FDG PET/MRI may offer improvement in diagnostic accuracy, particularly as MRI methodology develops further, and also offers timesaving as a single scan, performed on a single attendance.

Wider studies in this sphere, such as that of Martin et al. (2019), which did not look specifically at oesophageal cancer, suggested that these benefits could be realised, finding 18F-FDG PET/MRI facilitates staging comparable to that of 18F-FDG PET/CT and improves lesion detectability in selected cancers, potentially helping to promote fast, efficient local and whole-body staging in one step, when additional MRI is recommended. Their results confirmed the potential for a reduction of radiation exposure by using 18F-FDG PET/MRI instead of 18F-FDG PET/CT, which may be of relevance in younger patients presenting with early disease.

There are limited studies looking specifically at 18F-FDG PET/MRI in oesophageal cancer, with results broadly in agreement. Lee et al. (2014), in a retrospective study of sequential 18F-FDG PET/MRI of 19 patients with non-metastatic oesophageal cancer, found that although 18F-FDG PET /MR is inferior to EUS, it was feasible for the identification of oesophageal wall layers, which was limited for CT and impossible for 18F-FDG PET/CT, suggesting 18F-FDG PET/MRI offers a diagnostic benefit for patients with oesophageal cancer in whom EUS is not suitable or appropriate. Our study did not include EUS results, as although EUS is conventionally included as part of the staging process for patients with localised tumours, as only 4/22 participants enrolled underwent an EUS, highlighting the number of patients for whom EUS may not be suitable.

Moving beyond diagnostics, 18F-FDG PET/MRI may also be useful in response evaluation. In a pilot study using 18F-FDG PET/MRI to evaluate the response of neoadjuvant therapy to predict resectability in patients with gastro-oesophageal junction adenocarcinoma, Belmouhand et al. (2019) found 18F-FDG PET/MRI response evaluation to be highly sensitive when predicting resectability, suggesting 18F-FDG PET/MRI may have a future role in this scenario.

### Limitations

Endoscopic ultrasound and PET/CT are the standard staging investigations for oesophageal cancer. We were not able to include EUS as a comparative modality as many of the participants were ineligible for EUS due having an obstructing tumour which the EUS scope cannot traverse. The majority of the cancers included on this study were locally advanced (T3 or T4), and it is thought that EUS may not add additional information in some cases of locally advanced oesophageal cancers (DaVee et al. 2017).

Second, the small sample size in this exploratory study limits the generalisability of these findings. We also included a heterogeneous population of adenocarcinoma, squamous cell and small cell carcinoma. Although this is reflective of our clinical practice, our numbers are not large enough to allow subgroup analysis to compare the modalities across different oesophageal cancer subtypes.

Third, all participants underwent 18F-FDG PET/CT first followed by 18F-FDG PET/MRI. It is known that many malignant lesions will continue to increase target-to-background 18F-FDG uptake with delayed imaging (Chan et al. 2011; Kumar et al. 2005) and the absence of randomisation may bias the outcome of the comparison in favour of higher sensitivity for 18F-FDG PET/MRI.

Fourth, the majority of our participants underwent neoadjuvant therapy prior to surgery (if they went on to have surgery), limiting direct comparison with a contemporaneous histopathological reference standard for T and N status. This is a fundamental limitation of this study, as the reference standard (tumour board consensus staging) is based on standard investigations rather than histopathology and therefore is influenced by the results of contrast-enhanced CT and 18F-FDG PET/CT.

## Conclusion

Our preliminary study has shown that 18F-FDG PET/MRI is a comparable modality to 18F-FDG PET/CT for oesophageal/gastro-oesophageal TNM staging. However, 18F-FDG PET/MRI does capture additional information, such as additional metastases, which may be of further clinical value.

## Supplementary Information

**Additional file 1**. Appendix 1. WBMRI Imaging Protocol – PET/MRI Oesophagus.

## Data Availability

Not applicable.

## Abbreviations

18F-FDG PET/CT: ^18^F-fluorodeoxyglucose positron emission tomography/computed tomography
18F-FDG PET/MRI: ^18^F-fluorodeoxyglucose positron emission tomography magnetic resonance imaging
TNM staging: Tumour–nodes–metastasis staging
SUVmax: Maximum standardised uptake value
ADC: Apparent diffusion coefficient
EUS: Endoscopic ultrasound

## References

[CR1] Belmouhand M (2019). Early response evaluation of neoadjuvant therapy with PET/MRI to predict resectability in patients with adenocarcinoma of the esophagogastric junction. Abdom Radiol.

[CR2] Chan W (2011). Dual-time-point 18F-FDG-PET/CT imaging in the assessment of suspected malignancy. J Med Imag Radiat on.

[CR3] DaVee T, Ajani JA, Lee JH (2017). Is endoscopic ultrasound examination necessary in the management of esophageal cancer?. World J Gastroenterol.

[CR4] Enzinger PC, Mayer RJ (2003). Esophageal Cancer. N Engl J Med.

[CR5] Kelly S (2001). A systematic review of the staging performance of endoscopic ultrasound in gastro-oesophageal carcinoma. Gut.

[CR6] Kumar R, Loving VA, Chauhan A, Zhuang H, Mitchell S, Alavi A (2005). Potential of dual-time-point imaging to improve breast cancer diagnosis with (18)F-FDG PET. J Nucl Med Off Publ Soc Nucl Med.

[CR7] Lee G (2014). Clinical implication of PET/MR imaging in preoperative Esophageal Cancer Staging: comparison with PET/CT, endoscopic ultrasonography, and CT. J Nucl Med.

[CR8] Linder G, Korsavidou-Hult N, Bjerner T, Ahlström H, Hedberg J (2019). 18F-FDG-PET/MRI in preoperative staging of oesophageal and gastroesophageal junctional cancer. Clin Radiol.

[CR9] Lordick F, Mariette C, Haustermans K, Obermannová R, Arnold D, Committee EG (2016) Oesophageal cancer: ESMO Clinical Practice Guidelines for diagnosis, treatment and follow-up. Ann Oncol 27(suppl_5): v50–v57. doi:10.1093/annonc/mdw329 10.1093/annonc/mdw329 27664261

[CR10] Martin O et al (2019) PET/MRI versus PET/CT in whole-body staging: results from a unicenter observational study in 1003 subsequent examinations. J Nucl Med 61(8): jnumed.119.233940. doi:10.2967/jnumed.119.233940 10.2967/jnumed.119.233940 31806777

[CR11] Pennathur A, Gibson MK, Jobe BA, Luketich JD (2013). Oesophageal carcinoma. Lancet.

[CR12] Quint LE, Glazer GM, Orringer MB (1985). Esophageal imaging by MR and CT: study of normal anatomy and neoplasms. Radiology.

[CR13] Rice TW, Patil DT, Blackstone EH (2017). 8th edition AJCC/UICC staging of cancers of the esophagus and esophagogastric junction: application to clinical practice. Ann Cardiothorac Surg.

[CR14] Riddell AM (2006). Potential of surface-coil MRI for staging of Esophageal Cancer. Am J Roentgenol.

[CR15] Riddell AM, Allum WH, Thompson JN, Wotherspoon AC, Richardson C, Brown G (2007). The appearances of oesophageal carcinoma demonstrated on high-resolution, T2-weighted MRI, with histopathological correlation. Eur Radiol.

[CR16] Riddell AM, Davies DC, Allum WH, Wotherspoon AC, Richardson C, Brown G (2007). High-resolution MRI in evaluation of the surgical anatomy of the Esophagus and posterior mediastinum. Am J Roentgenol.

[CR17] Ringheim A, de Neto GCC, Martins KM, Vitor T, da Cunha ML, Baroni RH (2018) Reproducibility of standardized uptake values of same-day randomized 68Ga-PSMA-11 PET/CT and PET/MR scans in recurrent prostate cancer patients. Ann Nucl Med 32(8): 523–531. doi: 10.1007/s12149-018-1275-7 10.1007/s12149-018-1275-7 29982989

[CR18] Sjoquist KM (2011). Survival after neoadjuvant chemotherapy or chemoradiotherapy for resectable oesophageal carcinoma: an updated meta-analysis. Lancet Oncol.

[CR19] Takashima S (1991). Carcinoma of the esophagus: CT vs MR imaging in determining resectability. Am J Roentgenol.

[CR20] van Rossum PSN et al (2015) Imaging of oesophageal cancer with FDG-PET/CT and MRI. Clin Radiol 70(1): 81–95. doi:10.1016/j.crad.2014.07.017 10.1016/j.crad.2014.07.017 25172205

[CR21] van Hagen P et al. (2012) Preoperative chemoradiotherapy for Esophageal or Junctional Cancer. N Engl J Med 366(22): 2074–2084. doi:10.1056/nejmoa1112088 10.1056/NEJMoa1112088 22646630

[CR22] van Vliet EPM, Heijenbrok-Kal MH, Hunink MGM, Kuipers EJ, Siersema PD (2008) Staging investigations for oesophageal cancer: a meta-analysis. Br J Cancer 98(3): 547–557. doi:10.1038/sj.bjc.6604200 10.1038/sj.bjc.6604200 PMC2243147 18212745

