# Supplementary material for: Initial experience in staging primary oesophageal/gastro-oesophageal cancer with 18F-FDG PET/MRI
Source: Eur J Hybrid Imaging. 2021 Dec 13;5:23. doi: 10.1186/s41824-021-00117-y (PMC8666393; doi:10.1186/s41824-021-00117-y)
Supplement: Supplementary file 1 — Additional file 1. Appendix 1. WBMRI Imaging Protocol – PET/MRI Oesophagus. [file 41824_2021_117_MOESM1_ESM.docx]

**Appendix 1**

| WBMRI Imaging Protocol – PET/MRI Oesophagus | | | | |  |
| --- | --- | --- | --- | --- | --- |
|  | | | | |  |
| **Image contrast** | **T1-W** | **DWI (b-values 50 & 900 s/mm2)** | **T2-W** | **T1-W post-contrast** | **PET**  **(FDG)** |
| Sequence | DIXON 3D FLASH | DW-EPI | HASTE | DIXON 3D FLASH (Caipi-3) |  |
| Imaging plane | Axial | Axial | Axial | Axial | Axial |
| Number of slices per imaging station | 40 | 40 | 40 | 60 |  |
| Acquired Slice Thickness (mm) | 5 | 5 | 5 | 2 |  |
| Reconstructed Slice Thickness (mm) | 5 | 5 | 5 | 3.83 | 2 |
| Slice gap (mm) | 0 | 0 | 0 | 0 |  |
| FOV (mm) | 430 | 430 | 430 | 380 | 718 |
| Acquired voxels (mm x mm) | 1.92 x 1.34 | 3.16 x 3.16 | 1.92 x 1.34 | 1.58 x 1.19 |  |
| Reconstructed matrix | 320 | 136 | 320 | 320 | 171 |
| Reconstructed voxels (mm x mm) | 0.7 x 0.7 | 1.6 x 1.6 | 0.7 x 0.7 | 0.6 x 0.6 | 4.1 x 4.1 |
| Phase-encoding direction | AP | AP | AP | AP |  |
| TR (ms) | 4.02 | 8800 | 700 | 5.22 |  |
| TE (ms) | TE1 = 1.23,TE 2 = 2.46 | 85 | 107 | TE1 = 2.46,TE2 = 3.69 |  |
| Flip angle / ° | 10 | 90 | 90; refocusing angle 153 | 9 |  |
| Number of signal averages | 1 | 4 | 1 | 1 |  |
| Fat suppression | N/A | STIR | None | N/A |  |
| Acquisition Time (per station) | 15s | 3min 58s | 2 x 14s | 14s | 4min |
